# The Role of V-Set Ig Domain-Containing 4 in Chronic Kidney Disease Models

**DOI:** 10.3390/life13020277

**Published:** 2023-01-19

**Authors:** Sang Youb Han, Jung Yeon Ghee, Jin Joo Cha, Young Sun Kang, Han Seong Kim, Dae Young Hur, Dae Ryong Cha

**Affiliations:** 1 Department of Internal Medicine, Inje University, Ilsan-Paik Hospital, Goyang 10380, Republic of Korea; 2 Department of Internal Medicine, Korea University, Ansan Hospital, Ansan 15355, Republic of Korea; 3 Department of Pathology, Inje University, Ilsan-Paik Hospital, Goyang 10380, Republic of Korea; 4 Department of Anatomy and Tumor Immunology, Inje University College of Medicine, Busan 47392, Republic of Korea

**Keywords:** VSIG4, kidney, UUO, doxorubicin, podocyte

## Abstract

V-set Ig domain-containing 4 (VSIG4) regulates an inflammatory response and is involved in various diseases. However, the role of VSIG4 in kidney diseases is still unclear. Here, we investigated VSIG4 expression in unilateral ureteral obstruction (UUO), doxorubicin-induced kidney injury mouse, and doxorubicin-induced podocyte injury models. The levels of urinary VSIG4 protein significantly increased in the UUO mice compared with that in the control. The expression of VSIG4 mRNA and protein in the UUO mice was significantly upregulated compared with that in the control. In the doxorubicin-induced kidney injury model, the levels of urinary albumin and VSIG4 for 24 h were significantly higher than those in the control mice. Notably, a significant correlation was observed between urinary levels of VSIG4 and albumin (r = 0.912, *p* < 0.001). Intrarenal VSIG4 mRNA and protein expression were also significantly higher in the doxorubicin-induced mice than in the control. In cultured podocytes, VSIG4 mRNA and protein expressions were significantly higher in the doxorubicin-treated groups (1.0 and 3.0 μg/mL) than in the controls at 12 and 24 h. In conclusion, VSIG4 expression was upregulated in the UUO and doxorubicin-induced kidney injury models. VSIG4 may be involved in pathogenesis and disease progression in chronic kidney disease models.

## 1. Introduction

Various mechanisms are involved in the pathogenesis of chronic kidney disease (CKD) including an inflammatory response, the activation of the renin–angiotensin–aldosterone system, and hyperfiltration [1,2]. Currently, blockades of the renin–angiotensin–aldosterone system have been used to attenuate the progression of CKD; however, they have failed to completely prevent disease progression. As renal fibrosis is the final pathologic change in most CKDs, anti-fibrotic treatment is a promising option. However, in most clinical trials, renal fibrosis has not been successfully halted [3,4]

V-set Ig domain-containing 4 (VSIG4) is a B7 superfamily and one of the complement receptors primarily expressed in macrophages [5]. VSIG4 regulates the inflammatory response by the binding components of the complement pathway. It also negatively regulates T cell proliferation [6]. A new role of VSIG4 has recently been reported. Its expression increases in several malignant tumors and promotes the epithelial–mesenchymal transition (EMT) in several malignant diseases such as lung cancer and glioblastoma [7,8].

However, the role of VSIG4 in kidney diseases has been scarcely reported, and the reported results are conflicting. An inflammatory response was aggravated in a VSIG4 knock-out unilateral ureteral obstruction (UUO) model [9]. However, VSIG4 is related to the EMT under latent membrane protein-1 and the high-glucose condition in human tubular epithelial cells [10,11]. Considering that the EMT is one of the main mechanisms of renal fibrosis, VSIG4 may be related to kidney diseases. To investigate the role of VSIG4 in CKDs, in this study, we investigated the expression of VSIG4 in UUO- and doxorubicin-induced kidney injury mouse models, which are known animal models of renal fibrosis. In addition, VSIG4 expression was determined in doxorubicin-stimulated podocytes, which are some of the most important intra-glomerular cells and a treatment target for focal segmental glomerular sclerosis [12,13].

## 2. Materials and Methods

### 2.1. Animal Studies

To develop the UUO model, 8-week-old male *C57/BL6* mice (*n* = 20) were allocated to two equal groups for 14 days: sham and UUO groups. The left ureter was ligated at two points, the upper and middle ureters, and was cut between them. To develop the doxorubicin-induced kidney injury model, 6-week-old male *BALB/c* mice were randomly assigned to control (*n* = 9) and doxorubicin groups (*n* = 9). Thereafter, 11 mg/kg of doxorubicin (Sigma, St. Louis, MO, USA) diluted with 0.9% saline was administered to the doxorubicin group, and the same volume of isotonic saline was administered to the control mice via the tail vein. All mice were fed with a standard food tap water under a constant temperature of approximately 23 °C and a humidity of approximately 55% with a 12-:12-h light–dark cycle. The urine of mice in metabolic cages was collected for 24 h before sacrificing the mice. All mice were anesthetized with tribromoethanol (avertin 50 mg/kg, i.p.). All kidney tissues were collected and stored at –80 °C until further analysis. Albumin (ALPCO, Westlake, OH, USA) and VSIG4 (Bioassay Technology Laboratory, Shanghai, China) levels in the urine were determined using an enzyme immunosorbent assay with commercial kits according to the manufacturers’ instructions.

### 2.2. Podocyte Culture

To evaluate the effect of doxorubicin on VSIG4 expression, cultured mouse podocytes were used as previously described [14]. Briefly, podocytes were cultured at 33 °C until they attained confluency and incubated at 37 °C for 10–14 days to allow cell differentiation. After serum deprivation (1.0%) for 24 h, the sub-confluent cells were treated with doxorubicin at 1.0 or 3.0 μg/mL. All cells, cultured in triplicates, were harvested at 12 or 24 h.

### 2.3. Quantitative Real-Time Polymerase Chain Reactions

The total RNA was extracted using the TRIzol reagent and cDNA was prepared using SuperScript™ II Reverse Transcriptase (Invitrogen, Carlsbad, CA, USA) as previously described [14]. The levels of target genes were examined using quantitative real-time polymerase chain reaction (RT-PCR) using a SYBR premix kit (Roche Diagnostics, Indianapolis, IN, USA). After initial heating for 10 min at 50 °C and 5 min at 95 °C, 22–30 cycles were conducted for 10 s at 95 °C and 30s at 60 °C. The relative levels of gene expression were normalized to the level of *β-actin* mRNA expression. The sequences of primers used were as follows: *VSIG4* (sense: 5’-TCCCTGGCTTCCTTTCTTCT-3’; antisense: 5’-CCAAACCCAGGATTTCTCAA-3’) and *β-actin* (sense: 5’-GGACTCCTATGTGGGTGACG-3’; antisense: 5’-CTTCTCCATGTCGTCCCAGT-3’).

### 2.4. Immunohistochemical Staining for VSIG4

For immunohistochemical staining, 4 µm kidney sections were prepared. For antigen retrieval, the sections were heated at 80 °C for 30 min in 10 mmol/L citrate buffer solution at pH 6.0. Endogenous peroxidase blocking was conducted in 3.0% H_2_O_2_ in methanol for 20 min. Further blocking was conducted in normal goat serum at room temperature for 20 min. The sections were then incubated overnight at 4 °C with mouse polyclonal anti-VSIG4 (1:200; R&D system, Minneapolis, MS, USA). The sections were stained using the anti-goat HRP-DAB cell and tissue staining kit (R&D system) and counterstained with Mayer’s hematoxylin. Negative tissue controls were prepared under identical conditions with buffer solution and without primary antibodies. For VSIG4 immunostaining, four scores were semi-quantitatively assigned based on the extent of positive glomerular cells and tubulointerstitial fields: 0, absent or <25% positive area; 1, 25–50% positive area; 2, 50–75% positive area; and 3, >75% positive area.

### 2.5. Western Blot Analysis

Samples for Western blotting were prepared with podocytes and kidney cortex using commercial reagents (NE-PER Nuclear and Cytoplasmic Extraction Reagents, Thermo Scientific, Rockford, IL, USA). A 4–15% gradient gel was used to separate 10 μg of protein. Transferred membranes were probed with antibodies against VSIG4 (rabbit polyclonal, 1:500; Abcam Inc., Cambridge, MA, USA) or β actin (mouse monoclonal, 1:10,000; Sigma-Aldrich, St. Louis, MO, USA) overnight at 4 °C. After incubation with an HRP-labeled secondary antibody (1:1000), the signal was visualized using an enhanced chemiluminescence method (Amersham, Buckinghamshire, UK). An HRP-linked secondary antibody was used at a 1:1000 dilution. Amershm ECL detection reagents (Buckinghamshire, UK) were used to visualize the signals.

### 2.6. Statistical Analysis

Statistical analyses were performed using SPSS software version 25 (IBM Corp, New York, NY, USA). The statistical significance of differences was analyzed using *Student’s t-test or the Mann–Whitney U test*. We used Spearman’s test to analyze the correlation between the level of urinary albumin and VSIG4. Results with *p* < 0.05 were considered as statistically significant.

## 3. Results

### 3.1. UUO Model

One mouse in the UUO group died before euthanasia and was thus not included in the analysis. Finally, the control (*n* = 10) and UUO mice (*n* = 9) were analyzed on day 14. The level of urinary albumin (mg/mgCr) was not significantly different between the groups: 23.9 ± 15.7 vs. 40.2 ± 40.7, *p* = 0.33.

**Figure 1:**
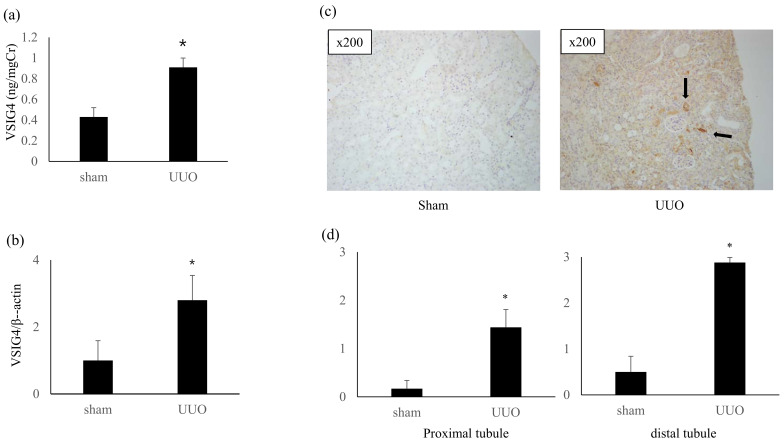
The expression of VSIG4 in the UUO model. (**a**) Urinary VSIG4 level. The values are presented as a ratio of VSIG4 to urinary creatinine concentrations. (**b**) *VSIG4* mRNA expression in the renal cortex. (**c**) Representative immunohistochemical staining of VSIG4. Diffuse VSIG4 immunostaining was observed in the proximal and distal tubules. Strong cytoplasmic VSIG4 staining was found focally in some atrophic tubules (arrow). (**d**) Semiquantitative expression of VSIG4 in the renal tubules. * *p* < 0.05 vs. sham.

The levels of urinary VSIG4 protein (ng/mgCr) increased in the UUO mice compared with that in the control mice: 0.92 ± 0.08 vs. 0.44 ± 0.09, *p* = 0.001 (Figure 1).

The intrarenal VSIG4 mRNA expression was significantly upregulated in the UUO mice compared with that in the control (2.8 ± 0.74 vs. 1.0 ± 0.18, *p* = 0.02). The results of immunohistochemical staining of VSIG4 were consistent with the VSIG4 mRNA expression: distal tubule, 2.88 ± 0.11 vs. 0.50 ± 0.0.34, *p* < 0.001; proximal tubule, 1.44 ± 0.37 vs. 0.16 ± 0.16, *p* = 0.02 (Figure 1). Diffuse but weak VSIG4 immunostaining was observed in the proximal and distal tubules. Strong cytoplasmic VSIG4 staining was observed focally in some atrophic tubules.

### 3.2. Doxorubicin-Induced Kidney Injury Model

#### 3.2.1. Baseline Characteristics

**Figure 2:**
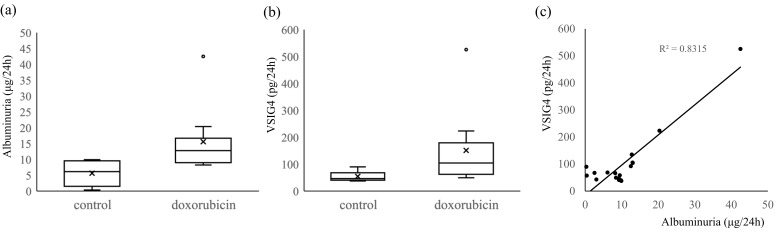
Urinary levels of albumin and VSIG4 in the doxorubicin-induced kidney injury mouse model. Urinary levels of albumin (**a**) and VSIG4 (**b**) for 24 h were significantly higher in the doxorubicin group than in control, respectively (*p* < 0.05). The levels were different between the groups analyzed using non-parametric analysis. (**c**) Correlation between albuminuria and urinary VSIG4 levels.

The urine volume (mL) was higher in the doxorubicin-induced kidney injury mice group than in the control: 2.16 ± 0.22 vs. 1.04 ± 0.001, *p* = 0.015. The level of albuminuria (μg) in the doxorubicin-treated mice was higher than that in the control mice: median, 12.7 (IQR, 9.48–13.03) vs. 6.12, (IQR 2.56–9.26), *p* = 0.006 (Figure 2).

#### 3.2.2. VSIG4 Expression

The urinary VSIG4 protein (pg) expression for 24 h measured using ELISA was also higher in the doxorubicin-induced mice group than in the control group (median, 104.1 [IQR 66.3–135.1] vs. 46.3 [IQR 40.9–67.2], *p* = 0.006). Notably, a close correlation was observed between urinary levels of VSIG4 and albumin (r = 0.912, *p* < 0.001) (Figure 2).

**Figure 3:**
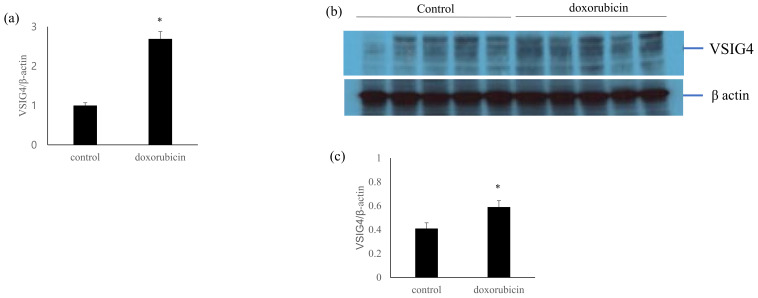
Intrarenal VSIG4 expression in the doxorubicin-induced kidney injury animal model. (**a**) Intrarenal VSIG4 mRNA using quantitative RT-PCR; (**b**) representative VSIG4 expression determined using Western blotting. (**c**) Semiquantitative expression of VSIG4. Values are expressed as mean ± SE. *: *p* < 0.05 vs. control.

The mRNA level of VSIG4 in the doxorubicin-treated mice was 2.69-fold higher than that in the control group. Western blotting was performed to confirm the expression of VSIG4 protein. The protein expression was significantly upregulated in the doxorubicin-induced kidney injury mouse group (Figure 3).

### 3.3. Doxorubicin-Induced Podocyte Injury Model

**Figure 4:**
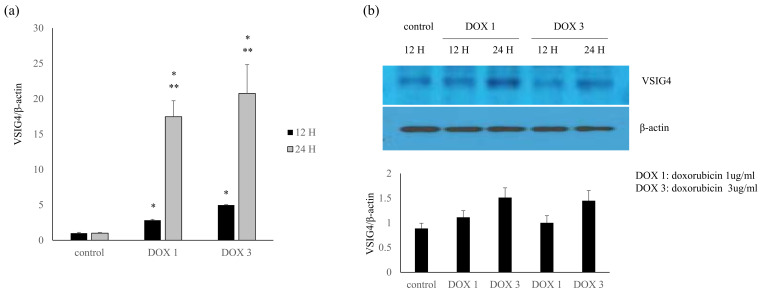
VSIG4 expression in doxorubicin-stimulated podocytes. (**a**) *VSIG4* mRNA level determined using quantitative RT-PCR; (**b**) VSIG4 protein level determined using Western blotting. Values are expressed as mean ± SE. *: *p* < 0.05 vs. control, **: *p* < 0.05 vs. 12 h.

We evaluated the effect of doxorubicin on VSIG4 expression in cultured podocytes. Doxorubicin significantly upregulated VSIG4 expression in a time- and dose-dependent manner (Figure 4). The *VSIG4* mRNA expression was significantly higher in doxorubicin-treated (1.0 and 3.0 μg/mL) podocytes than in the controls at 12 and 24 h. The mRNA expression showed a 20.7-fold increase in the doxorubicin (3.0 μg/mL) group at 24 h. The pattern of protein expression, determined using Western blot analysis, was similar to that of mRNA expression.

## 4. Discussion

This study demonstrated the upregulation of VSIG4 expression in animal and in vitro models of CKD. Urinary and intrarenal VSIG4 levels were significantly increased in both UUO and doxorubicin-induced kidney injury mouse models. Furthermore, the level of urinary VSIG4 was highly correlated with the level of urinary albumin in the doxorubicin-induced kidney injury mouse model. Furthermore, VSIG4 mRNA and protein expressions were upregulated in cultured podocytes stimulated with doxorubicin. These results suggest that VSIG4 might play a role in the pathogenesis of CKD.

The doxorubicin-induced nephropathy model is a well-characterized kidney injury animal model of CKD [12,13]. We found that VSIG4 expression was upregulated and that it correlated with the urinary albumin level in the doxorubicin-induced kidney injury animal model. However, VSIG4 immunostaining was unsuccessful. Alternatively, we confirmed higher VSIG4 expressions using Western blotting. In addition, we evaluated podocytes stimulated with doxorubicin, as doxorubicin-induced nephropathy is characterized by podocyte injury followed by glomerulosclerosis, which mimics focal segmental glomerular sclerosis [12,13]. VSIG4 expression was evidently upregulated in doxorubicin-treated podocytes. Considering these results, VSIG4 plays an important role in doxorubicin-induced kidney injury models.

Both UUO and doxorubicin-induced kidney injury animal models are representative CKD models of tubular and glomerular damage, respectively. Fibrosis is a common finding in both models with advanced renal dysfunction. Considering that inflammation and fibrosis are major findings in UUO models, high VSIG4 expression in damaged tubules in the UUO model indicates the role of VSIG4 in the progression of kidney injury in UUO models. In contrast, in the *VSIG4* knock-out UUO model, intrarenal inflammatory infiltrations were reportedly significantly greater in the VSIG4 −/− mice than in the VSIG4 +/+ mice [9]. This result implies that VSIG4 could attenuate an inflammatory reaction in the UUO model. However, in this study, the CD68 level was not different between the wild-type and knockout mice, although VSIG4 was dominantly expressed in the macrophages. Considering that VSIG4 regulates T-cell proliferation and macrophage function, the systemic effect of *VSIG4* deletion could affect these results. However, the role and effect of VSIG4 on kidney injury remain unclear and the systemic and local effects of VSIG4 must be further clarified in the future studies.

A few studies have analyzed the role of VSIG4 in kidney disease models. VSIG4 is reportedly upregulated in a diabetic kidney model. Bioinformatic analyses have revealed that VSIG4 expression is upregulated in diabetic nephropathy [15,16]. VSIG4 was selected as one of the seven complement cascade genes among 32 hub genes. Its expression was upregulated in patients with diabetes and its levels negatively correlated with GFR [16]. We found that VSIG4 expression was related to the EMT pathway under the condition of high glucose and Epstein–Barr virus-related protein overexpression in proximal tubular epithelial cells [10,11]. In addition, VSIG4 expression was apparently upregulated in a type 2 diabetic animal model [unpublished data]. These previous and present study findings suggest that VSIG4 might play a role in the fibrotic progression of kidney diseases.

This study has some limitations. First, the origin of urine in the UUO model was the contralateral kidney. Second, the immunostaining of VSIG4 was not available in the doxorubicin-induced kidney injury mouse model. Finally, the role of VSIG4 could not be clarified with the current models.

## 5. Conclusions

VSIG4 expression was upregulated in the kidney injury animal model. Considering previous study results on the diabetic kidney disease model together with the present study results, VSIG4 could be an important mediator of kidney injury. Further studies are required to clarify the role of VSIG4 in kidney diseases.

## Data Availability

The data of the current study are available from the corresponding author upon reasonable request.
